# Chemistry: General Medical and Pharmaceutical, Including the Chemistry of the United States Pharmacopœia

**Published:** 1871-06

**Authors:** 


*Chemistry: General Medical and Pharmaceutical, Including the Chemistry of the United States Pharmacopœia. A Manuel on the General Principles of the Science and their Applications to Medicine and Pharmacy.* By JOHN ATTFIELD, Ph. D. F. C. S., Professor of Practical Chemistry to the Pharmaceutical Society of Great Britain, etc., etc. HENRY C. LEA, Philadelphia, 1871.

---

This work, of 550 pages, is from the Second London edition, revised by the author. Being devoted, in a good degree, to the consideration of pharmaceutical operations, and the chemistry of officinal therapeutic agents, it seems to be designed for the use of Medical Students. The modernized system of nomeclature has been adopted, and will, doubtless, soon become popular with pharmaceutists.

The want of time, just now, to give this book an examination necessary to make a full review of it, compels us to postpone further notice of it for a future number of the Journal.

The work is for sale at the book-store of M. Lynch & Co., Whitehall street, Atlanta, Ga.

